# Life cycle environmental emissions and health damages from the Canadian healthcare system: An economic-environmental-epidemiological analysis

**DOI:** 10.1371/journal.pmed.1002623

**Published:** 2018-07-31

**Authors:** Matthew J. Eckelman, Jodi D. Sherman, Andrea J. MacNeill

**Affiliations:** 1 Department of Civil and Environmental Engineering, Northeastern University, Boston, Massachusetts, United States of America; 2 Department of Anesthesiology, Yale School of Medicine, New Haven, Connecticut, United States of America; 3 Department of Surgery, University of British Columbia, Vancouver, Canada; University of Wisconsin, Madison, UNITED STATES

## Abstract

**Background:**

Human health is dependent upon environmental health. Air pollution is a leading cause of morbidity and mortality globally, and climate change has been identified as the single greatest public health threat of the 21st century. As a large, resource-intensive sector of the Canadian economy, healthcare itself contributes to pollutant emissions, both directly from facility and vehicle emissions and indirectly through the purchase of emissions-intensive goods and services. Together these are termed life cycle emissions. Here, we estimate the extent of healthcare-associated life cycle emissions as well as the public health damages they cause.

**Methods and findings:**

We use a linked economic-environmental-epidemiological modeling framework to quantify pollutant emissions and their implications for public health, based on Canadian national healthcare expenditures over the period 2009–2015. Expenditures gathered by the Canadian Institute for Health Information (CIHI) are matched to sectors in a national environmentally extended input-output (EEIO) model to estimate emissions of greenhouse gases (GHGs) and >300 other pollutants. Damages to human health are then calculated using the IMPACT2002+ life cycle impact assessment model, considering uncertainty in the damage factors used. On a life cycle basis, Canada’s healthcare system was responsible for 33 million tonnes of carbon dioxide equivalents (CO_2_e), or 4.6% of the national total, as well as >200,000 tonnes of other pollutants. We link these emissions to a median estimate of 23,000 disability-adjusted life years (DALYs) lost annually from direct exposures to hazardous pollutants and from environmental changes caused by pollution, with an uncertainty range of 4,500–610,000 DALYs lost annually. A limitation of this national-level study is the use of aggregated data and multiple modeling steps to link healthcare expenditures to emissions to health damages. While informative on a national level, the applicability of these findings to guide decision-making at individual institutions is limited. Uncertainties related to national economic and environmental accounts, model representativeness, and classification of healthcare expenditures are discussed.

**Conclusions:**

Our results for GHG emissions corroborate similar estimates for the United Kingdom, Australia, and the United States, with emissions from hospitals and pharmaceuticals being the most significant expenditure categories. Non-GHG emissions are responsible for the majority of health damages, predominantly related to particulate matter (PM). This work can guide efforts by Canadian healthcare professionals toward more sustainable practices.

## Abstract 2

Andrea MacNeill and colleagues model for the lifetime environmental emissions from the Canadian healthcare system, showing that in saving lives, it also contributes to air pollution and health damages.

## Author summary

**Why was this study done?:**

- Global environmental change is being increasingly recognized as a threat to human health.
- Paradoxically, the delivery of healthcare services has a significant environmental footprint, which contributes to an increasing global burden of disease.

**What did the researchers do and find?:**

- We used Canadian healthcare expenditures from 2009 to 2015 to estimate the amount of greenhouse gas and other pollutant emissions generated by Canadian healthcare activities.
- Using a model that links environmental emissions to disease, we were able to estimate the damages to human health caused by the Canadian healthcare system.
- We found that Canadian healthcare activities generated 33 million tonnes of greenhouse gas emissions and over 200,000 tonnes of other pollutant emissions, resulting in 23,000 disability-adjusted life years lost annually.

**What do these findings mean?:**

- The Canadian healthcare system is a significant contributor to national greenhouse gas emissions, primarily due to emissions from hospitals, pharmaceuticals, and physician services.
- Healthcare professionals should be leaders in climate change mitigation, as this is a major public health issue, and should advocate for environmentally sustainable change within the healthcare industry.
- Bottom-up studies of specific healthcare activities are required to identify strategies for improving the environmental footprint of the health sector.

## Introduction

Among high-income countries, most environmentally mediated death and disability are due to cardiovascular disease, respiratory illness, and cancer. These are all epidemiologically linked to air pollution, which is the single largest driver of environmentally mediated mortality, responsible for one in eight deaths globally [1,2]. In Canada alone, it is estimated that more than 20,000 premature deaths each year are attributable to air pollution [3]. In addition, global health risks from climate change are rapidly rising. Climate change is presently responsible for an estimated 141,000 deaths globally each year from changing patterns of infectious diseases, food and water scarcity, and extreme weather events. Climate change has been identified as a major public health priority of the 21st century [4–6], as it is projected to result in an additional 250,000 deaths annually between 2030 and 2050 [5].

Paradoxically, pollutant emissions associated with the health sector itself adversely affect human health. Healthcare pollution stems both from direct activities, such as energy-intense hospital operations, as well as from indirect activities linked to healthcare, such as procurement and waste management [7–10]. Healthcare also contributes environmental hazards in the manufacture and disposal of pharmaceuticals and biohazardous products [11,12], including inhaled anesthetics that are themselves potent greenhouse gases (GHGs) [13–15]. Understanding the environmental performance of healthcare itself is therefore crucial to inform safety and quality improvement efforts [16–18]. There are growing calls internationally to measure and mitigate healthcare pollution, including by the World Health Organization [19], The World Bank [10], The Lancet Commission on Climate Change and Health [4], the Institute of Medicine [20], and Health Canada [21,22].

Health Canada is committed to sustainable development, understood as “meeting the needs of the present without compromising the ability of future generations to meet their own needs” [22]. Under the Federal Sustainable Development Act (FSDA) of 2008, Health Canada is required to measure and mitigate its own environmental footprint, including emissions contributing to climate change and air quality [21,22]. The FSDA requires that select government agencies prepare, implement, and report on departmental sustainable development strategies every 3 years, with the goal of reducing GHG emissions from federal government operations by 40% by 2030.

Health sector life cycle environmental emissions have been estimated on a national scale for three other countries in order to identify hot spots and prioritize mitigation efforts. Healthcare was found to contribute 3%–4% of national GHG emissions in the UK [8] and 7% in Australia [23]. A similar study for the US found that the health sector emits 9%–10% of GHGs nationally, but also considered other types of emissions, with similar proportional results for emissions of particulate matter (PM) and ozone precursors [7,24]. In total, these US healthcare-related emissions result in an estimated annual loss of 614,000 disability-adjusted life years (DALYs), the only such national damage estimate to date [7,24]. Building on our prior work on the US health sector [7,24], our objective is to estimate the life cycle emissions and associated environmental impacts and secondary health damages of the Canadian healthcare system. Comparative analyses among countries allow us to investigate structural differences in healthcare services and the environmental performance of upstream industries, while within Canada the results may guide strategic performance improvement efforts in alignment with Health Canada’s commitment to sustainable development.

## Methods

Life cycle emissions and consequent health damages of Canada’s healthcare sector were estimated using standard input-output life cycle assessment (IO-LCA) techniques, which model the natural resources required and pollutants emitted throughout the life cycle of a good or service (its use plus supply chain) on the basis of how much that good or service costs. Thus, IO-LCA models use monetary expenditures as their primary input. The IO-LCA approach is in widespread use around the world and forms the basis for consumption-based environmental accounting, such as calculating embodied energy and carbon at national and international levels. For example, Environment Canada reports national GHG emissions from fuel combustion from both production and consumption perspectives, relying on IO-LCA modeling results in order to account for emissions from Canadian consumption [25].

While the IO-LCA approach is well established, the integration of healthcare expenditures with economic and environmental data for Canada is novel. IO-LCA models track flows among different sectors of a national or international economy and are constructed from a series of tables, each of which represents different types of flows into and out of an economic sector. For the present study, the IO-LCA model we used for Canada includes two main components: (1) economic, showing monetary flows among Canada’s economic sectors, and (2) emissions, showing physical flows from each of Canada’s economic sectors in the form of pollutant releases. The economic portion of the IO-LCA model was built from provincial or national input-output accounts by Statistics Canada. These economic tables were then extended by adjoining data on emissions from each industry sector from Statistics Canada Environmental Accounts and the Canadian National Pollutant Release Inventory to create environmentally-extended input-output (EEIO) tables. The EEIO tables were then manipulated using standard economic input-output assessment techniques [26,27] in order to link final demand (in the form of healthcare expenditures) to pollutant emissions throughout the economy as a response to that demand, according to

$$b\;=\;R{\left(I-A\right)}^{-1}y,$$

where ***b*** is a vector life cycle emissions of pollutants to air, water, or soil; ***R*** is a matrix of pollutant emissions per dollar of economic output from each economic sector;(***I*** − ***A***)^−**1**^ is the Leontief inverse matrix, where ***A*** is the matrix of intersectoral monetary flows and ***I*** is the identity matrix; and ***y*** is the final demand vector of health expenditures.

Finally, deleterious impacts on human health and environmental quality are estimated by multiplying each pollutant’s life cycle emission by its so-called characterization factor, which represents its fate, potential exposure, and the effect of that exposure, based on physical-chemical and toxicological data. Each data source and calculation step is further detailed in the paragraphs that follow.

Comprehensive expenditures in Canada’s healthcare sector were gathered from the National Health Expenditures (NHEX) database maintained by the Canadian Institute for Health Information (CIHI) [28]. Data were extracted for the years 2009–2015; 2015 is the last full reporting year available at the time of writing. Expenditures in current dollars were deflated to constant Canadian dollar (C$) 2009, based on Consumer Price Index (CPI) rates from Statistics Canada. NHEX expenditures are reported in 13 broad categories, two of which (“Hospitals” and “Other Institutions”) were further disaggregated into public and private expenditures, shown in Table 1.

**Table 1:** Mapping between NHEX health expenditure categories and Statistics Canada/Open IO-Canada commodity codes.

NHEX Category	Open IO Commodity Code
Hospitals (Private)	Hospital services (fees)
Hospitals (Public)	Hospital services provided by governments
Other Institutions (Private)	Nursing and residential care services
Other Institutions (Public)	Residential care facility services provided by governments
Physicians	Physician services
Dental Services	Dental services
Vision Care Services	Other health practitioner services
Other	Other health practitioner services
Prescribed Drugs	Pharmaceutical and medicinal products
Nonprescribed Drugs	Pharmaceutical and medicinal products
Capital	Nonresidential building construction
Public Health	Other health and social assistance services
Administration	Average of (1) accident and sickness insurance services and (2) other provincial and territorial government services
Health Research	Research and development services
Other	Average of (1) ambulatory healthcare services provided by Non-Profit Institutions serving Households, (2) medical, dental, and personal safety supplies, instruments, and equipment, and (3) nursing and residential care services

NHEX methodology notes: https://www.cihi.ca/sites/default/files/document/nhex-methodological-notes_2016_en.pdf.

Abbreviations: IO, input-output; NHEX, National Health Expenditures.

Expenditures for each category (the final demand vector ***y***) are then linked with the EEIO model. Here, we use the Open IO-Canada model developed by the International Reference Centre for the Life Cycle of Products, Processes and Services (CIRAIG) at École Polytechnique de Montréal. Details of the model formulation have been published elsewhere [29]. This EEIO model is made of linked spreadsheets that track monetary flows associated with 238 commodities (goods and services) exchanged across 112 industry sectors, as well as emissions per C$ of expenditure. The entire model is open and freely downloadable, and all commodities, industry sectors, and substances are listed on the project website [29].

NHEX expenditures were matched with Open IO-Canada commodity codes, as shown in Table 1. Life cycle pollutant emissions from healthcare activities ***b*** were calculated by multiplying C$ 2009 expenditures with life cycle emissions intensity values (in kg emission/C$1 million) for each emission and each sector, derived from the Open IO-Canada model. Emissions to air, water, and soils are considered separately, comprising 318 discrete emission flows in total. Emissions are then added across results for each expenditure category to arrive at the total quantity for each pollutant, occurring throughout the Canadian economy, associated with national healthcare expenditures.

The environmental and public health impacts of these emissions are modeled using the IMPACT2002+ life cycle impact assessment model [30], whose list of covered pollutants matches that employed by the Open IO-Canada model. Pollutants are classified according to the types of environmental impacts they cause and expressed against a reference substance. For example, GHG emissions are calculated as carbon dioxide equivalents (CO_2_e) by multiplying the total quantities of carbon dioxide, methane, and nitrous oxide emitted to air by their 100-year global warming potentials (GWP_100_) of 1, 21, and 310, respectively. The GWP_100_ values for methane and nitrous oxide have been since updated to 28 and 265, respectively, but we use the original model values for internal consistency (other GHGs such as sulfur hexafluoride are not included in the Open IO-Canada model).

Total health damages are calculated by multiplying each emission by its corresponding end point characterization factor. These characterization factors are derived in the IMPACT2002+ model for several impact categories representing distinct damage pathways, including cancer and non-cancer toxicity, disease from exposure to respirable organics and inorganics (PM), and skin cancer from ozone layer depletion. Emissions are then linked to health damages using so-called characterization factors that account for pollutant fate and transport, exposure, and effects and are expressed in health damages per unit of emission for each substance and receiving compartment (air, water, or soil). For the four impact categories stemming from direct exposures (cancer and non-cancer toxicity, disease from respirable organics and inorganics), the characterization factor is a product of a pollutant’s fate (typically derived over a large region), exposure and intake (inhalation and ingestion), and effect (disease risk and severity). Pollutant fate is estimated using a nested box model accounting for partitioning and intermedia transfer (e.g., via deposition), leading to a change in pollutant concentration in air, water, soils, and food. Exposure is modeled to occur through air inhalation, water ingestion, food ingestion, and incidental soil ingestion. The fraction of pollutant emitted that is subsequently inhaled or ingested is called the intake fraction (kg_*intake*_/kg_*emitted*_). The intake fraction is then multiplied by a linearized dose-response function slope factor (risk of disease incidence per kg_*intake*_) and a disease severity factor (DALYs per disease case) to arrive at the end point characterization (damage) factor (DALYs/kg_*emitted*_).

There is significant uncertainty in these direct exposure damage factors, arising from uncertainty in each constituent fraction of fate, exposure, toxicity, and severity. The IMPACT2002+ model and its updates have included different emission scenarios, for example, to low- and high-population areas, in order to provide ranges of results. Here, we use the widest range of damage factors available for each impact category from the most recent version of IMPACT2002+ [31]. Respirable organics do not have a range of damage factors provided and so are assumed to vary over two orders of magnitude from the single value provided. For the remaining impact categories of ozone depletion and global warming, the damage pathways are based on indirect effects from environmental changes caused by the emitted substances. The characterization factor for ozone depletion is based on the ozone depletion potential (ODP) of each pollutant, and causally linked to the incidence of skin cancer due to reduced absorption of ultraviolet (UV) radiation by the stratospheric ozone layer. Future health damages from GHG emissions are not explicitly considered in the IMPACT2002+ model. Instead, we consider a range of health damage factors derived for similar impact assessment models, as detailed in previous work [24], which vary over three orders of magnitude, largely depending on the path of global GHG emissions in the coming decades.

Additional uncertainties associated with the economic and environmental data reported by Canada’s statistics and environment agencies, the fixed economic structure of EEIO models, and the matching of NHEX expenditure categories with Open IO-Canada commodity codes are discussed separately in the Study limitations section.

The impact assessment is conducted at a national aggregate scale and no attempt is made to apply the results to subpopulations, locations, or specific individuals. Healthcare life cycle emissions are compared to national totals using published values from Environment and Climate Change Canada over the same time period [32]. Health damages from all emissions are compared to US estimates from the same period [7,24]. No such damage estimates exist for the UK or Australia, precluding comparison.

## Results

### GHG emissions

Canada’s healthcare life cycle GHG emissions, including both emissions directly from healthcare facilities and from their supply chains, increased from 29.6 million metric tonnes (Mt) CO_2_e in 2009 to 33.0 Mt CO_2_e in 2014, or 4.6% of the national GHG emissions total. Over the same period, monetary health expenditures as a share of national gross domestic product (GDP) have varied between 10.9% and 11.6%.

Disaggregated results for GHG emissions by healthcare expenditure category are provided in Table 2. In 2015, the most important expenditure categories were Public Hospitals (22%), Prescribed Drugs (21%), and Physicians (13%). Grouping Prescribed and Non-prescribed Drugs together as pharmaceuticals represents 25% of total life cycle healthcare GHG emissions, making it the largest expenditure category by emissions, just ahead of Hospitals (Public and Private together represent 24%).

**Table 2:** Absolute healthcare life cycle GHG emissions (million Mt CO_2_e) by NHEX category for 2009–2015.

NHEX category	GHG emissions intensity (tonnes CO_2_e/million C$)	2009	2010	2011	2012	2013	2014	2015
Hospitals (Private)	135	0.7	0.7	0.7	0.7	0.7	0.8	0.8
Hospitals (Public)	135	6.5	6.8	7.0	7.0	7.1	7.1	7.1
Other Institutions (Private)	132	0.7	0.7	0.7	0.7	0.8	0.8	0.9
Other Institutions (Public)	81.4	1.1	1.2	1.2	1.2	1.2	1.2	1.3
Physicians	142	3.7	3.9	4.0	4.1	4.2	4.3	4.4
Dental Services	142	1.7	1.7	1.6	1.7	1.7	1.7	1.8
Vision Care Services	142	0.6	0.5	0.5	0.5	0.5	0.5	0.6
Other	142	0.4	0.4	0.4	0.5	0.5	0.5	0.6
Prescribed Drugs	253	6.3	6.9	6.8	6.7	6.7	6.8	7.0
Nonprescribed Drugs	253	1.2	1.2	1.2	1.2	1.2	1.2	1.3
Capital	284	2.5	2.8	2.7	2.7	2.3	2.3	2.4
Public Health	165	1.6	1.6	1.7	1.7	1.7	1.8	1.9
Administration	123	0.7	0.7	0.7	0.7	0.7	0.7	0.6
Health Research	121	0.4	0.4	0.4	0.4	0.4	0.4	0.4
Other	44.7	1.6	1.7	1.7	1.7	1.7	1.8	2.0
**Total**	**162**	**29.6**	**31.2**	**31.4**	**31.5**	**31.4**	**32.0**	**33.0**
CA TOTAL GHGs*		682	694	700	707	716	716	714
% of CA TOTAL GHGs		4.3%	4.5%	4.5%	4.5%	4.4%	4.5%	4.6%
% of CA TOTAL GDP**		11.6%	11.6%	11.3%	11.3%	11.0%	10.9%	11.4%

*Canada GHG Inventory.

**NHEX Data.

Abbreviations: CA, Canada; CO_2_e, carbon dioxide equivalent; GDP, gross domestic product; GHG, greenhouse gas; Mt, metric tonne; NHEX, National Health Expenditures.

**Fig 1:**
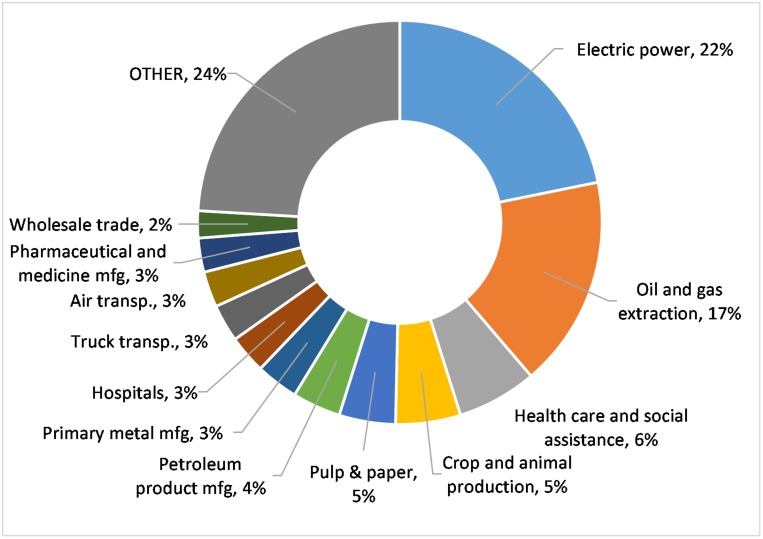
Relative percent contributions of economic sectors to Canadian healthcare life cycle GHG emissions based on 2015 expenditures. GHG, greenhouse gas; mfg, manufacturing; transp., transportation.

Fig 1 shows results disaggregated by the economic sectors that contribute to emissions, rather than by the health expenditures that represent final demand. The top 12 sectors are shown, each of which contributes 2% or more to healthcare GHG emissions. The top two sectors are energy sectors that contribute most to Canada’s national GHG emissions, namely electricity generation (primarily from combustion) and oil and gas extraction (primarily from wells and pipelines). Direct emissions from healthcare activities and facilities can be seen from the healthcare and social assistance (6.4%) and hospitals (3.1%) economic sectors, which, along with nursing and residential care (0.4%), represent just one tenth of healthcare-related GHG emissions, with indirect emissions from upstream economic activities contributing the other approximately 90%. Interestingly, agriculture is another significant contributor (5.2%), primarily from nitrous oxide emissions from fertilizers and methane from ruminant animals, underscoring the interlinkages between the healthcare system and the food system.

### Environmental impacts and health damages

Carbon dioxide is by far the largest emission by mass, but Canada’s healthcare system also generated life cycle emissions of more than 210,000 metric tonnes of pollutant emissions other than CO_2_ to air, water, and soils, some of them hazardous to humans. The largest emissions to air were for ammonia, carbon monoxide, methanol, nitrogen oxides, non-methane volatile organic compounds (NMVOCs), PM, and sulfur dioxide. Top emitted substances by mass are shown in Table 3, with the percentage of the national total emitted, when tracked.

**Table 3:** Top 15 life cycle emissions by mass (excluding carbon dioxide) from 2015 Canadian healthcare expenditures.

Emitted to	Substance	Emissions (tonnes)	Percent Canada total
Air	Sulfur dioxide	80,000	6.6%
Air	Carbon monoxide	46,000	0.8%
Air	Nitrogen oxides	44,000	2.3%
Air	NMVOC, unspecified origin	13,000	
Air	Particulates, >2.5 um, and <10 um	6,700	-
Air	Particulates, <2.5 um	3,600	-
Water	Nitrate	3,600	-
Water	Ammonia	3,300	-
Air	Particulates, >10 um	2,900	-
Air	Ammonia	1,200	0.3%
Air	Methanol	1,100	-
Air	Hydrogen chloride	440	-
Air	Sulfuric acid	380	-
Water	Phosphorus	380	-
Air	2-Propanol	350	-

Abbreviation: NMVOC, non-methane volatile organic compound.

Health damages from non-GHG pollutant emissions attributable to Canadian healthcare totaled 14,700 DALYs in 2015 (Table 4), with an uncertainty range of 4,100–26,000 DALYs per year. Damage factors with uncertainty ranges are also listed for each corresponding reference substance in Table 4. The great majority (>90%) of DALYs from non-GHG pollutants are due to respiratory disease stemming from exposure to PM respiratory inorganics. Health damages from exposure to PM have been extensively reported and are the largest contributor to air pollution-related morbidity and mortality worldwide [2]. Indirect damages from global climate change effects such as malnutrition, spread of infectious disease, and temperature extremes may contribute an additional 8,410 DALYs annually, but this value ranges from 373 up to 581,000 DALYs, depending on the future global GHG emissions scenario and associated health damage factor that is assumed [24]. The wide range of reported values reflects the high uncertainty associated with this estimate.

**Table 4:** Health damages (DALYs) from 2015 Canadian healthcare expenditures.

NHEX category	Climate Change	Respiratory Inorganics	Respiratory Organics	Carcinogens	Noncarcinogens	Ozone Layer Depletion
Reference compound	CO_2_	PM_2.5_	C_2_H_4_	C_2_H_3_Cl	C_2_H_3_Cl	CFC-11
Damage factor (DALYs/kg emission of reference compound)	2.60 (0.10–176) × 10^−7^	7.0 (2.0–12) × 10^−4^	2.1 (0.2–21) × 10^−6^	3.2 (0.5–5.8) × 10^−5^	2.6 (0.4–4.7) × 10^−6^	1.8 (0.2–18) × 10^−3^
Hospitals (Private)	199 (9–13,700)	310 (87–520)	0 (0–4)	15 (2–27)	6 (1–10)	0 (0–3)
Hospitals (Public)	1,820 (81–126,000)	2,800 (800–4,810)	4 (0–37)	136 (23–249)	50 (9–92)	3 (0–30)
Other Institutions (Private)	219 (10–15,100)	300 (86–520)	0 (0–4)	17 (3–32)	6 (1–12)	0 (0–3)
Other Institutions (Public)	326 (14–22,500)	490 (140–840)	1 (0–7)	32 (5–58)	11 (2–20)	1 (0–7)
Physicians	1,130 (50–77,900)	1,600 (440–2,700)	2 (0–19)	85 (14–160)	31 (5–57)	1 (0–13)
Dental Services	458 (20–31,600)	630 (180–1,100)	1 (0–8)	35 (6–64)	13 (2–23)	1 (0–5)
Vision Care Services	148 (7–10,200)	200 (58–350)	0 (0–2)	11 (2–21)	4 (1–7)	0 (0–2)
Other	154 (7–10,700)	210 (61–360)	0 (0–3)	12 (2–21)	4 (1–8)	0 (0–2)
Prescribed Drugs	1,790 (79–124,000)	3,300 (930–5,600)	6 (1–64)	97 (16–180)	37 (6–67)	9 (1–92)
Nonprescribed Drugs	320 (14–22,100)	580 (170–1,000)	1 (0–11)	17 (3–32)	7 (1–12)	2 (0–16)
Capital	601 (27–41,500)	1,500 (420–2,500)	1 (0–13)	110 (18–200)	41 (7–75)	1 (0–13)
Public Health	475 (21–32,800)	640 (180–1,100)	1 (0–8)	33 (6–60)	12 (2–23)	1 (0–5)
Administration	162 (7–11,200)	210 (60–360)	0 (0–3)	10 (2–19)	4 (1–7)	0 (0–2)
Health Research	102 (5–7,050)	130 (38–230)	0 (0–2)	8 (1–15)	3 (1–6)	0 (0–1)
Other	508 (22–35,000)	955 (270–1,600)	2 (0–15)	53 (9–97)	18 (3–34)	1 (0–10)
**Total DALYs**	**8,410 (373–581,000)**	**13,700 (3,900–23,500)**	**19 (1–200)**	**670 (110–1,200)**	**250 (43–450)**	**20 (1–204)**

Abbreviations: DALY, disability-adjusted life year; NHEX, National Health Expenditures.

Hospitals and Prescribed Drugs each contribute nearly a quarter of total health damages, while the Physicians category is the next largest contributor, at 12% of the total. Note that different expenditure categories do not have the same proportions of damage from each impact category. These different proportions reflect the different upstream economic sectors that are activated for each category of expenditure and their subsequent delivery. For example, Capital expenditures involve significant activity in building construction, including emissions from heavy equipment and the production of building materials such as steel and concrete, while Hospital expenditures involve significant activity in electricity production, including emissions from fossil fuel extraction and combustion.

## Discussion

### Summary of main findings

As a service sector, the Canadian healthcare system does not have substantial on-site emissions, as might a manufacturing sector such as iron and steel production. However, on a life cycle basis, including supply chain and waste treatment linkages, our work shows that healthcare activities are responsible for nationally significant quantities of GHGs and other pollutants. These life cycle emissions are estimated to cause between 4,100 and 23,000 DALYs of health damages for non-GHG pollutants, dominated by PM. An additional 373 to 581,000 DALYs are estimated for life cycle GHGs from healthcare, depending largely on the path of global GHG emissions in the coming decades. In keeping with findings in the US, UK, and Australia, expenditures in the Canadian healthcare system on Hospitals and Pharmaceuticals stand out for their significant contributions to emissions and health impacts, and efforts are underway, particularly in the UK, to measure these emissions in a more detailed, bottom-up fashion [33,34].

### Comparison to other countries

**Fig 2:**
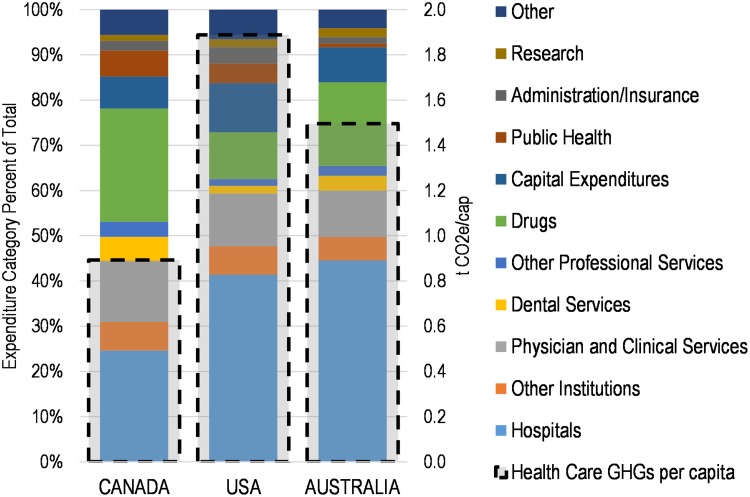
Relative contributions of expenditure categories to healthcare life cycle GHG emissions and absolute results per capita, 2014 results, Canada-United States-Australia. CO_2_e, carbon dioxide equivalent; GHG, greenhouse gas; Mt CO_2_e/cap, metric tonnes CO_2_e per capita.

As a proportion of national GHG emissions, Canada’s result of 4.6% is above that of the UK but well below those for Australia and the US [7,8,23]. The economic and accounting models used to create these estimates differ, and so comparative results among countries should be interpreted cautiously. A specific barrier to comparability is the different way that each country categorizes healthcare expenditures in their national accounts, with varying levels of detail or aggregation, and some unique categories depending on the structure of each healthcare system. Nevertheless, we have grouped expenditures into high-level categories to enable some comparison across national systems. These results are shown visually in Fig 2 for relative contributions per sector (left axis, in percentage) to the national total, and per capita absolute contributions (right axis, in tonnes CO_2_e/person). Numerical results are provided in tabular form in S1 and S2 Tables.

For the expenditure category breakdowns, two comparisons are particularly notable. First, compared to the US and Australia, Canada has proportionally lower life cycle GHG emissions for expenditures by Hospitals (26% versus >40% for the other two countries). This result may reflect on-site energy and emissions considerations such as greater winter heating needs and lower summer cooling needs. In addition, hospitals are generally electricity-intensive buildings that house extensive ventilation and electrical equipment [35], and GHG emissions from electricity generation vary significantly among the three countries, as discussed further below. Second, Canada has proportionally more emissions from Drugs (both prescribed and nonprescribed) than the other two countries (26% versus 10% for the US and 18% for Australia). This result has a number of potential drivers, including differences in drug pricing, relative proportions of elderly populations, trends in prescribing, as well as factors that affect the relative size of other expenditure categories, such as physicians’ salaries. Direct fuel and electrical energy used for drug manufacturing per unit mass of pharmaceuticals produced is unlikely to vary much among the three countries, but again the emissions associated with the generation of electricity used by manufacturers vary considerably. Other expenditure categories are also listed, although, particularly for these smaller categories, cross-country comparisons should be undertaken cautiously given the differences in reporting structures, expenditure category descriptions, and the different IO-LCA models employed in each country.

The healthcare life cycle GHG emissions per capita shown in Fig 2 reveal significant differences among the three countries, with the results for Canada (0.9 metric tonnes CO_2_e per capita [t CO_2_e/cap]) well below those for the US (1.9 t CO_2_e/cap) and Australia (1.5 t CO_2_e/cap). These total results reflect both the differences in per capita healthcare expenditures as well as the relative life cycle GHG emissions intensity of those expenditures.

Over the study period 2009–2015, absolute healthcare GHG emissions increased in Canada by about 10%, from 29.6 to 33.0 million metric tonnes CO_2_e. The trend in the US was also upwards but even more pronounced, increasing more than 9% just from 2009 to 2013 [7]. Several macro-trends are important to consider in interpreting this comparison. First, similar to the US, where national GHG emissions remained relatively stable (decreasing slightly) from 2009 to 2015, Canada’s national GHG emissions increased by just 5% over that time frame. Second, in both countries, real (inflation-adjusted) healthcare expenditures increased by 8%–10% over that period in absolute terms, but as a proportion of overall GDP, Canadian healthcare spending declined from 11.6% to 10.9% and then increased in 2015 to 11.4%, while in the US the proportion of GDP due to healthcare spending rose steadily from 17.3% to 17.7%, following a large jump of more than 1% during the global financial crisis of 2007–2008. Controlling for the proportion of healthcare spending, Canada’s healthcare system had a ratio of percent GDP expenditures to percent national GHG emissions of 2.5:1, whereas the US system had a ratio of 1.8:1, meaning that the Canadian system is less carbon intensive, emitting fewer life cycle GHGs per dollar (US$ or C$) spent.

In both Canada and the US, the largest contributing economic sector to healthcare GHG emissions is electricity generation, transmission, and distribution (as seen in Fig 1). One important reason for the relative difference in healthcare life cycle GHG emissions between the two countries is that electricity generation in Canada is less carbon intensive than in the US, with the majority (60%) of generation from hydroelectricity, resulting in estimated 2015 GHG emissions factor of 130 g CO_2_e/kWh, versus 470 g CO_2_e/kWh for the more fossil fuel-based electricity in the US [32,36].

### Comparison to other Canadian industrial sectors

As noted above, as a proportion of national totals, the Canadian healthcare sector is responsible for 11.4% of expenditures but only 4.6% of GHG emissions, such that its GHG emissions intensity is much less than the national average. This is expected for service sectors, which have relatively high value-added and typically low direct emissions from operations [27]. GHG emissions intensities are listed in Table 1 by expenditure category, with an average of 162 tonnes CO_2_e/million C$ of expenditures. Because of the physical resource intensity of healthcare (buildings, equipment, supplies, electricity, etc.), its emissions intensity is high compared to high-cost service sectors such as “Legal Services” (18 tonnes CO_2_e/million C$) or administrative service sectors such as “Life, Accident, and Sickness Insurance” (97 tonnes CO_2_e/million C$). It is also higher than other resource-intensive services such as “Primary and Secondary School Education” (108 tonnes CO_2_e/million C$). In contrast, the GHG emissions intensity of Canadian healthcare is much lower than those of basic agricultural or manufacturing sectors with relatively low value-added and typically high direct emissions, such as “Grains and Oilseed” (2,150 tonnes CO_2_e/million C$), “Iron Ore Mining” (418 tonnes CO_2_e/million C$), or ‘Petrochemicals’ (382 tonnes CO_2_e/million C$) [29].

### Valuing health damages from healthcare

Health damages associated with pollution can be expressed in economic terms. In the case of GHGs, one such measure is the social cost of carbon (SCC), which is an estimate of the monetized damages associated with an incremental increase in carbon emissions, specifically including environmentally mediated disease and disability, as well as other projected effects of climate change. Environment and Climate Change Canada currently estimates the value of one tonne of CO_2_ emissions at C$41. This value reflects not only the anticipated cost of increased GHG emissions but also the cost savings associated with avoided emissions, which would be disproportionately recovered by the health sector. The Lancet Countdown to 2030, a working group focusing on this pivotal time frame, has identified the pricing of health externalities of fossil fuels as an important economic incentive to accelerate progress on climate change and health. For example, the International Monetary Fund (IMF) has estimated that implementation of nationally appropriate energy prices that incorporate health impacts could cut ambient air pollution deaths by approximately one third and reduce GHG emissions by more than 20% [37]. Four Canadian provinces currently have some form of carbon price. By 2018, all provinces will be obligated to ensure a minimum price on carbon that will increase to a targeted C$50/tonne as of 2022. While carbon pricing will incentivize decarbonization of domestic goods and services, it does not address emissions from imported items, and therefore further action is likely required.

### Study limitations

This national-level study used aggregated data and multiple modeling steps to link healthcare expenditures to emissions to health damages. Each step in this chain of causality carries uncertainty. This analysis focused on uncertainty in the damage characterization factors that represent the fate-exposure-effect link between emissions of a pollutant to its effects on human health. Considering the link between expenditures and emissions, both NHEX expenditures and the Open IO-Canada model rely on national economic and environmental accounts that are compiled by government agencies, with potential uncertainty in both the magnitude and classification of economic and environmental flows. Another important source of uncertainty is the fixed structure of the EEIO model, which reflects the Canadian economy as it existed in 2009. Input-output tables published by national statistical agencies are by their nature retrospective, often with a 5+ year lag for the full tables. It is typical practice to deflate expenditures to an earlier model year in order to conduct IO-LCA modeling [27]. However, care must be taken when interpreting results to consider how Canada’s economic structure (represented in the monetary flow tables) and environmental performance (represented in the adjoined emissions table) have changed in the intervening period. One approach is to stochastically perturb both the ***A*** (economic) and ***R*** (environmental) matrices using Monte Carlo simulation and derive confidence intervals from the distribution of results, as was done in a recent study for Australia’s healthcare system [23]. Additional modeling limitations include imperfect matching between NHEX health expenditure categories and EEIO model sectors and the application of generic IMPACT2002+ life cycle characterization factors to model Canada-specific impacts. Geographic and temporal representativeness of modeling parameters can be improved through impact assessment model upgrades and updates to improve relevance and accuracy, underscoring the importance of revisiting these estimates of life cycle emissions and healthcare damages as better estimates of damage characterization factors become available. Finally, a last important study limitation is the aggregated nature of both NHEX data and EEIO model sectors, which precludes any in-depth analysis of specific subsector health sector activities.

### Conclusion and future directions

The 2015 Lancet Commission on Climate and Health appealed to governments and to the health community to address “the greatest global health opportunity of the 21st century” [38]. National-level studies of health sector emissions bring attention to the contribution of the healthcare industry to global climate change and pollution, providing a baseline against which to measure performance improvement and allowing for participation in both voluntary and mandatory climate mitigation mechanisms. The UK was the first country to report a national-level health system carbon footprint in 2009, with published updates every 2–3 years [8]. By instituting this measurement and reporting structure, National Health Service (NHS) England has been able to demonstrate an 11% reduction in GHG emissions from healthcare activities from 2007 to 2015 despite increased utilization of health services during this time. With this improved performance, NHS England is meeting short-term targets and moving toward a goal of 80% emissions reduction by 2050, as established by the UK Climate Change Act of 2008. With increasing methodological standardization, it is expected that measuring and reporting national-level health sector emissions will also allow for useful comparisons between countries that can inspire widespread improvements in environmental performance.

One key recommendation from the Lancet Commission is the empowerment of health professionals to take a leadership role in health promotion through environmental protection and, specifically, to investigate the environmental impacts of healthcare activities. While the current study has examined this on a macroscale, numerous other studies have investigated individual products, processes, and communities of care in order to identify actionable areas for improved environmental performance within healthcare systems [14,39–41].

The healthcare community is uniquely positioned to advocate for pollution mitigation policies and practices that will reduce the global burden of disease, thereby improving the health of current and future generations. No industry is better positioned to benefit from the synergies of health and environmental sustainability. As healthcare systems struggle to meet the demands of ageing populations and increasing complexity of care, improved population health would lessen the burden of disease and ease the financial strain. The governance structure of the Canadian healthcare system lends itself to these sorts of cross-sectoral public policy-making, bringing together health, economics, and sustainable development in order to ensure a continued high standard of living for generations to come.

## Supporting information

S1 Table Relative contributions of expenditure categories to healthcare sector GHG emissions, 2014, Canada-United States-Australia. GHG, greenhouse gas.
(DOCX)

S2 Table Healthcare life cycle greenhouse gas emissions per capita, 2014 data, Canada-United States-Australia. (DOCX)

## Abbreviations

C$: Canadian dollar
CA: Canada
CIHI: Canadian Institute for Health Information
CIRAIG: International Reference Centre for the Life Cycle of Products, Processes and Services
CO_2_e: carbon dioxide equivalent
CPI: Consumer Price Index
DALY: disability-adjusted life year
EEIO: environmentally extended input-output
FSDA: Federal Sustainable Development Act
GDP: gross domestic product
GHG: greenhouse gas
GWP_100_: 100-year global warming potential
IMF: International Monetary Fund
IO: input-output
IO-LCA: input-output life cycle assessment
mfg: manufacturing
Mt: metric tonne
Mt CO2e/cap: metric tonnes CO2e per capita
NHEX: National Health Expenditures
NHS: National Health Service
NMVOC: non-methane volatile organic compound
ODP: ozone depletion potential
PM: particulate matter
SCC: social cost of carbon
transp: transportaition
UV: ultraviolet
