# Supplementary material for: Life cycle environmental emissions and health damages from the Canadian healthcare system: An economic-environmental-epidemiological analysis
Source: PLoS Med. 2018 Jul 31;15(7):e1002623. doi: 10.1371/journal.pmed.1002623 (PMC6067712; doi:10.1371/journal.pmed.1002623)
Supplement: S1 Table — GHG, greenhouse gas. (DOCX) [file pmed.1002623.s001.docx]

**S1 Table. Relative contributions of expenditure categories to health care sector GHG emissions, 2014, Canada-USA-Australia.**

| **CANADA** | | **UNITED STATES** | | **AUSTRALIA** | |
| --- | --- | --- | --- | --- | --- |
| **High-level category**  **NHEX categories** | **Emissions,**  **% of total** | **High-level category**  **NHE categories** | **Emissions,**  **% of total** | **High-level category**  **IHW categories** | **Emissions,**  **% of total** |
| Hospitals | 26% | Hospitals | 41% | Hospitals | 45% |
| Hospitals (Public) | 23% | Hospital Care | 36% | Public hospitals | 34% |
| Hospitals (Private) | 2% | Durable Medical Equipment | 3% | Private hospitals | 10% |
|  |  | Other Non-Durable Medical Products | 2% |  |  |
| Other Institutions | 7% | Other Institutions | 6% | Other Institutions | 5% |
| Other Institutions (Private) | 3% | Nursing Care Facilities and Continuing Care Retirement Communities | 6% | Community health and other | 5% |
| Other Institutions (Public) | 4% |  |  |  |  |
| Physician and Clinical Services | 14% | Physician and Clinical Services | 12% | Physician and Clinical Services | 10% |
| Physicians | 14% | Physician and Clinical Services | 12% | Referred medical services (specialists) | 6% |
|  |  |  |  | General practice | 4% |
| Dental Services | 6% | Dental Services | 2% | Dental Services | 3% |
| Other Professional Services | 3% | Other Professional Services | 1% | Other Professional Services | 2% |
| Vision Care Services | 2% | Other Professional Services | 1% | Other health practitioners | 2% |
| Other | 2% |  |  |  |  |
| Drugs | 26% | Drugs | 10% | Drugs | 18% |
| Prescribed Drugs | 22% | Prescription Drugs | 10% | Benefit-paid pharmaceuticals | 9% |
| Non-Prescribed Drugs | 4% |  |  | All other medications | 9% |
| Capital Expenditures | 7% | Capital Expenditures | 11% | Capital Expenditures | 8% |
| Capital | 7% | Structures and Equipment | 11% | Capital expenditure (buildings) | 8% |
| Public Health | 6% | Public Health | 4% | Public Health | 1% |
| Administration/Insurance | 2% | Administration/Insurance | 4% | Administration/Insurance | 1% |
|  |  | Government Administration | 2% |  |  |
|  |  | Net Cost of Health Insurance | 1% |  |  |
| Research | 1% | Research | 2% | Research | 2% |
| Other | 2% | Other | 7% | Other | 4% |
|  |  | Home Health Care | 3% | Aids and appliances | 3% |
|  |  | Other Health, Residential, and Personal Care | 4% | Patient transport services | 1% |

National health expenditure categories from Canada National Health Expenditure Database (NHEX), US National Health Expenditure Data (NHE), Australian Institute of Health and Welfare (IHW)
