# Supplementary material for: Life cycle environmental emissions and health damages from the Canadian healthcare system: An economic-environmental-epidemiological analysis
Source: PLoS Med. 2018 Jul 31;15(7):e1002623. doi: 10.1371/journal.pmed.1002623 (PMC6067712; doi:10.1371/journal.pmed.1002623)
Supplement: S2 Table — (DOCX) [file pmed.1002623.s002.docx]

**S2 Table. Health Care Life Cycle Greenhouse Gas Emissions per Capita, 2014 data, Canada-USA-Australia**

|  | **CANADA** | **UNITED STATES** | **AUSTRALIA** |
| --- | --- | --- | --- |
| **Total Health Care Life Cycle GHG Emissions**  **(million metric tons)** | 32 | 614 | 36 |
| **Population (millions)** | 35.9 | 325.1 | 23.9 |
| **Per Capita Health Care Life Cycle GHG Emissions**  **(t CO2e/person)** | 0.9 | 1.9 | 1.5 |
